# Athletic Sports

**Published:** 1873-01

**Authors:** 


### ATHLETIC SPORTS.

There is a general impression that out-door games and competitive amusements, such as rowing, boating, sailing base ball, cricket, and the like, are beneficial to the health, growth, and constitution of the people. As practiced in this country, they are worse than useless; they imperil the health, debase the morals, and foster idle drunkenness and unthrift wherever they are practiced. All competitions of skill and agility with us are attended with bets—a plain phase of gambling—of getting something from your friend without any substantial return; it is simply a lottery; and all know that where lotteries most abound, the people are most degraded—are lazy, improvident, and thriftless; then the step to crime is short, direct, and speedily made.

Time is lost in productive labor—not only the time of the performers, but that of idlers—which all competitions draw. Besides, it is indisputable, that more ardent spirits are drank in consequence of every target excursion and base ball game and cricket matches and rowing, than if none of these had taken place. There is also more cursing and swearing, and angry altercation and quarrels, and fights and murders, than if all the parties had been at useful, productive labor at home. Loss of health and life is not an unfrequent attendant of these pastimes. R. E. C., one of the most promising of the Yale College graduates for 1872, recently died, as the result of some mishap in a gymnasium. It is said of him, that he was one of the finest writers in his class, and the best gymnast in college; it is suggestively stated that "his health had not been good for a long time." It must have required long practice to have become a model gymnast, and yet it failed to preserve his health, and failed to restore it.

A champion rower on the Hudson died recently, under thirty years of age.

The Greeks and Romans made games and competitions a national amusement, and about the time when these amusements reached their highest, and their system of public and private betting became most general, they began to decay as nations, in morals as well as in prowess.

A great deal has been said of the athletic sports of England. The peasantry haven't the time; the young men of the nobility have not the inclination; and on a critical examination, it may be found, that not one in ten of the middle classes ever indulges in these sports to any systematic extent. How these pastimes promote liquor drinking, gambling, and idleness, is open to all. How the health is lost, needs but short explanation. The competition produces exhaustion and excessive perspiration; in this condition, while the system is particularly sensitive to a cold, to cooling off too quickly, the powers of resistance against the ill effects of cold are reduced to a low point, and rheumatic affections and inflammations of a dangerous and fatal character are of not unfrequent occurrence.

It is well known that trained boxers seldom live long; the rigors of training seem to break down the constitution.

It is not denied that there are advantages to health and bodily vigor derived from wisely conducted gymnasiums, and from rowing, boating, swimming, and the like, when reasonable care is taken, and there is no competition, and no liquor drinking, and no fighting; but, without the beer or brandy, how many would go to a cricket, a rowing, or a target shooting?

The most advantageous form of exercise is that which is out of doors, which is steady, slow, continuous, and which brings pecuniary profit as a result. Ploughing takes precedence; next to that is chopping wood, splitting rails, hoeing corn—by the day! Then, there will be no danger of "over doing," of exhaustion, of excessive perspiration. If these forms of exercise are not available, walk an hour briskly every forenoon and every afternoon, living altogether on bread and butter, wheaten grits, fruits, berries, and grapes, at three regular times every day, and nothing whatever between. Any one who does this for two weeks will weigh more, will be stronger, healthier, and happier, than when he began.

